# Prevalence of Dyslipidemia in Patients With Type 2 Diabetes Mellitus: A Cross-Sectional Study

**DOI:** 10.7759/cureus.20222

**Published:** 2021-12-06

**Authors:** Hussain A Al Ghadeer, Mohammed Al Barqi, Abdullah Almaqhawi, Amal S Alsultan, Jinan A Alghafli, Murtada A AlOmaish, Zuhur A AlGhanem, Amer H Alsaqar, Abrar T Alatiyyah, Yasamiyan A Alburayh, Abdulatif AlOmair, Afaf I Almuhaysin, Ali A Alsaeed

**Affiliations:** 1 Paediatrics, Maternity and Children Hospital, Al-Ahsa, SAU; 2 Family and Community Medicine, King Faisal University, Al-Ahsa, SAU; 3 Family and Community Medicine, Medical University of Warsaw, Waesaw, POL; 4 Family and Community Medicine, Charles University, Charles, CZE

**Keywords:** hdl-c, ldl-c, diabetes, saudi arabia, dyslipidemia

## Abstract

Introduction: Diabetes mellitus (DM), a chronic metabolic noncommunicable disease (NCD), has assumed epidemic proportions worldwide. Type 2 diabetes (T2D) is defined as chronic high blood glucose levels due to the deficiency of insulin or resistance to it. Dyslipidemia is one of the major causes of cardiovascular diseases in patients with T2D. It is characterized by elevated plasma triglyceride (TG), low high-density lipoprotein (HDL) cholesterol, and high low-density lipoprotein (LDL) cholesterol, which is mostly present in patients with DM.

Methods: We conducted a retrospective cross-sectional study at the King Faisal University (KFU) Health Center in the eastern region of Saudi Arabia. The data of patients from October 2014 to February 2021 were collected. We collected the patients’ data from the KFU Health Center after obtaining approval from the KFU polyclinic administration. Prior ethical approval was taken from the Institutional Review Board of Ethics and Research Committee in the College of Medicine, KFU (approval number, 2020-10-62). We collected patients’ data, including their gender, age, nationality, and blood analysis (lipids profile - TGs, HDL, LDL, and hemoglobin A1c [HbA1c] levels).

Result: This study included 191 patients with T2D, 137 (71.7%) were from Saudi Arabia, and 54 (28.3%) were from other countries. Patients’ age ranged from 21 to 100 years, with a mean age of 56.2 ± 11.8 years. There were 107 (56%) females. Cholesterol levels ranged from 102 to 300 mg/dl with a mean value of 187.3 mg/dl.

Conclusion: In the current study, we assessed the association between HbA1c levels and lipid profiles in patients with T2D at the KFU Health Center in the eastern region of the Kingdom of Saudi Arabia. Our results on the adjusted relationship of HbA1c with lipid profile through regression model have demonstrated that HDL alone had significant adjusted relation with HbA1c if other factors are kept constant. We suggest focusing on TC and HDL levels in T2D health management in future studies.

## Introduction

Diabetes mellitus (DM), a chronic metabolic noncommunicable disease (NCD), has assumed epidemic proportions worldwide [1]. Type 2 diabetes (T2D) is defined as chronic high blood glucose levels due to the deficiency of insulin or resistance to it [2]. A report from the International Diabetes Federation Diabetes Atlas, 2020, has estimated approximately 463 million people with DM worldwide. Additionally, the number of patients with DM may reach 578 million by 2030 and 640 million by 2040 [3]. Several studies have reported the lack of awareness about the possible complications due to T2D in approximately half of the patients with (T2D) [3]. Chronic T2D can cause many complications, resulting in morbidity and mortality [4]. The complications that are caused by diabetes can be classified into two types: microscopic and macroscopic. The microscopic complications include retinopathy, neuropathy, and nephropathy; whereas, the macroscopic include cardiovascular diseases, peripheral artery disease, and stroke [5]. Additionally, many complications, such as dental disease, low immunity, might be caused because of T2D, but they cannot be included in the previous classification. Gestational diabetes can cause macrosomia in women. It is well established that a high body mass index is the major cause of insulin resistance and T2D [3].

Dyslipidemia is one of the major causes of cardiovascular diseases in patients with T2D. It is characterized by elevated plasma triglyceride (TG), low high-density lipoprotein (HDL) cholesterol, and high low-density lipoprotein (LDL) cholesterol, which is mostly present in patients with DM [6]. The TGs are involved in many individual molecular species, whereas lipoproteins are involved in many other lipid classes that contain multiple molecular species, the role of the individual molecular species in T2D remains unknown [7]. Hypercaloric and low fiber diets contribute to fat depositions in T2D that cause insulin resistance and lead to lipotoxicity [7]. The changes in lipid molecules in T2D are attributable to an increase in the level of fatty acid flux secondary to insulin resistance [8]. This research aimed to assess the prevalence of dyslipidemia among patients with T2D.

## Materials and methods

Research design

We conducted a retrospective cross-sectional descriptive study at the King Faisal University (KFU) Health Center in the eastern region of Saudi Arabia. The data of patients from October 2014 to February 2021 were collected.

Procedure

We collected the patients’ data from the KFU Health Center after obtaining approval from the KFU polyclinic administration. Prior ethical approval was taken from the Institutional Review Board of Ethics and Research Committee in the College of Medicine, KFU (approval number 2020-10-62). We collected patients’ data, including their gender, age, nationality, and blood analysis (lipids profile - TGs, HDL, LDL, and hemoglobin A1c [HbA1c] levels).

The inclusion criteria for the study were as follows: (i) age > 18 years; ​​​​​​​(ii) diagnosed with T2D for more than three months, ​​​​​​​(iii) on a regular follow-up with the KFU Health Center.

The exclusion criteria were as follows: ​​​​​​​(i) age < 18 years; ​​​​​​​(ii) diagnosed with T2D for less than three months; ​​​​​​​(iii) type 1 DM patients; ​​​​​​​(iv) has hypertension, on lipid-lowering drugs, and smoking habit; ​​​​​​​(v) had no coexisting disease associated with hyperlipidemia (e.g., hypothyroidism, renal disease, or hepatic disorders).

Study population

One hundred and ninety-one patients, who were suffering from T2D for at least one year, were included in our study.

Materials

At the KFU Health Center, the cut-off values for patients’ inclusion were as follows: HbA1c level, 4-5.9%; cholesterol, 100-200 mg/dl; HDL, 40-60 mg/dl; LDL, <100 mg/dl; TG, 37-150 mg/dl.

Statistical analyses

After collection, the data were modified, coded, and statistically analyzed using SPSS V22.0 (IBM SPSS, Inc., Chicago, IL, USA). All statistical analyses were performed using two-tailed tests. A P-value of <0.05 was considered statistically significant. Descriptive analysis was done for all categorical variables (personal data), based on the frequency and percent distribution, whereas mean with standard deviation and range described lipid profile and HbA1c level in total and by gender, comparing lipid profile and HbA1c by patients’ gender. Correlation analysis was used to assess the nature and strength of the unadjusted relationship between HbA1c and lipid profile, whereas a multiple linear regression model was used to assess the adjusted relation between HbA1c and lipid profile.

## Results

This study included 191 patients with T2D, 137 (71.7%) were from Saudi Arabia, and 54 (28.3%) were from other countries. Patients’ age ranged from 21 to 100 years, with a mean age of 56.2 ± 11.8 years. There were 107 (56%) females (Table 1).

**Table 1:** Personal characteristics of sampled T2D patients

Personal data	No (191)	%
Country		
Saudi Arabia	137	71.7%
Developing country	50	26.2%
Developed country	4	2.1%
Age in years		
20–30	5	2.6%
31–40	9	4.7%
41–50	35	18.3%
51–60	72	37.7%
61+	70	36.6%
Gender		
Male	84	44.0%
Female	107	56.0%

Table 2 exhibits lipid profile and glycosylated hemoglobin levels among patients with T2D in the Kingdom of Saudi Arabia. Cholesterol levels ranged from 102 to 300 mg/dl, with a mean value of 187.3 mg/dl. In total, 61 (32.3%) patients showed high cholesterol levels. HDL levels ranged from 25 to 91 mg/dl (mean value, 49.2 mg/dl). Forty (22.1%) patients with T2D showed high HDL levels. TG levels ranged from 56.3 to 616.5 mg/dl (mean value, 147 mg /dl); 64 (33.9%) patients had high levels of TGs. LDL values ranged from 33.5 to 195 mg/dl (mean, 105.2 mg/dl); 94 (57.7%) patients showed high LDL levels. HbA1c value ranged from 3.9% to 13% (mean, 8.1%); 174 (92.1%) patients with T2D had poor diabetic control (HbA1c > 5.9%).

**Table 2:** Lipid profile and glycosylated hemoglobin levels among T2D patients

Lipid profile and HbA1c	No	%	Range	Mean	SD
Cholesterol level (mg/dl)					
Normal	128	67.7%	102–300	187.3	34.7
High	61	32.3%			
High-density lipoprotein level (mg/dl)					
Normal	141	77.9%	25–91	49.2	13.5
High	40	22.1%			
Triglyceride (mg/dl)					
Normal	125	66.1%	56.3–616.5	147.0	74.8
High	64	33.9%			
Low-density lipoprotein level (mg/dl)					
Normal	69	42.3%	33.5–195.0	105.2	28.7
High	94	57.7%			
HbA1c level %					
Normal	15	7.9%	3.9–13.0	8.1	1.7
High	174	92.1%			

Table 3 illustrates gender-wise lipid profile and glycosylated hemoglobin levels among patients with T2D. Cholesterol level was insignificantly higher among female patients with T2D than among males (191.4 vs. 181.9 mg/dl; P = 0.062). HDL level among female patients was 54.2 mg/dl, whereas it was 42.9 mg/dl for male patients, with a statistically significant difference (P = 0.001). TG and LDL levels showed a slight difference among male and female patients. The average HbA1c level for female patients was 8.4%, whereas it was 7.7% for males (P = 0.008).

**Table 3:** Lipid profile and glycosylated hemoglobin levels among T2D patients by their gender *P-value less than 0.05

Lipid profile and HbA1c	Gender				P-value
	Male		Female		
	Mean	SD	Mean	SD	
Cholesterol (mg/dl)	181.9	35.0	191.4	34.0	0.062
High-density lipoprotein (mg/dl)	42.9	9.3	54.2	14.2	0.001*
Triglyceride (mg/dl)	151.3	83.8	143.7	67.3	0.580
Low-density lipoprotein (mg/dl)	106.6	32.4	104.1	25.7	0.487
HbA1c %	7.7	1.5	8.4	1.8	0.008*

Table 4 shows age-wise lipid profile and glycosylated hemoglobin levels among patients with T2D. Cholesterol level was insignificantly higher among patients with DM aged 40-60 years than among the older age group (189.4 vs. 184.4 mg/dl; P = 0.627). HDL among patients aged 20-39 years was 38.0 mg/dl in comparison to 52.3 mg/dl for patients aged >60 years with statistically significant difference (P = 0.002). TG levels were significantly higher among patients aged 20-29 years (mean, 210.6 mg/dl) than other patients aged >60 years (mean, 129 mg/dl) with P = 0.001. The average level of HbA1c among young patients (20-29 years) was 7.8% compared to 8.4% for patients aged >60 years (P = 0.186).

**Table 4:** Lipid profile and glycosylated haemoglobin levels among T2D patients by their age *P-value less than 0.05

Lipid profile and HbA1c	Age in years						P-value
	20–39		40–60		>60		
	Mean	SD	Mean	SD	Mean	SD	
Cholesterol (mg/dl)	185.3	33.4	189.4	35.5	184.4	33.9	0.627
High-density lipoprotein (mg/dl)	38.0	6.9	48.7	12.4	52.3	14.8	0.002*
Low-density lipoprotein (mg/dl)	108.1	30.7	105.9	31.8	103.2	22.1	0.803
Triglyceride (mg/dl)	210.6	145.3	150.8	72.0	129.0	50.3	0.001*
HbA1c %	7.8	1.1	7.9	1.7	8.4	1.9	0.186

Table 5 clarifies correlation analysis (among HbA1c lipid parameters) and linear regression analysis of T2D patients showing the dependency of HbA1c on other variables. HbA1c level showed significant positive intermediate crude correlation with cholesterol level (r = 0.25; P = 0.001), and HDL level (r = 0.23; P = 0.002); and it showed borderline significant positive correlation with LDL level (r = 0.14; P = 0.069). Adjusted relation of HbA1c with lipid profile through a regression model, considering all other factors constant showed that only HDL level had significant adjusted relation with HbA1c.

**Table 5:** Correlation analysis (between HbA1C lipid parameters) and linear regression analysis of T2D patients showing dependency of HbA1C on other variables *P-value less than 0.05

Lipid profile	Correlation analysis		Regression analysis	
	r	P-value	B	P-value
Cholesterol (mg/dl)	0.25	0.001*	0.03	0.979
High-density lipoprotein (mg/dl)	0.23	0.002*	0.29	0.033*
Low-density lipoprotein (mg/dl)	0.14	0.069	0.17	0.469
Triglyceride (mg/dl)	0.11	0.151	0.20	0.219

## Discussion

In the present study, the majority of the study sample was females. The higher number of females having T2D was conformed to the findings of other studies. This is explained by the effect of hormones, lower physical activities, and higher iron storage ability [9]. Additionally, diet and lifestyle play an important role in the control of glycemic levels.

In this study, the control rates of cholesterol, HDL, TG, LDL, and HbA1c for all patients with T2D were 67.7%, 77.9% 66.1%, 42.3%, 44.80%, and 7.9%, respectively. Lower control rates for HbA1c and LDL in patients with DM. A study from 2017 from China revealed a glycemic control rate of 30% in DM [10].

Our results showed that 61 (32.3%) patients had high cholesterol levels. HDL levels ranged from 25 to 91 mg/dl (mean value, 49.2 mg/dl); 40 (22.1%) patients with T2D had high HDL levels. TG values ranged from 56.3 to 616.5 mg/dl (mean, 147 mg/dl); 64 (33.9%) patients had high levels. LDL values ranged from 33.5 to 195 mg/dl (mean, 105.2 mg/dl); 94 (57.7%) patients had high LDL levels. Many prospective studies showed no association between lower HDL levels and the development of T2D in the future, although it affects the control of glycemic level [11]. We can also conclude that lower HDL is not helpful for glycemic control. Although Mullugeta et al. showed an association between high HbA1c and high TG, it did not show an association with LDL [12]. However, few studies showed a similar result of a positive correlation between HbA1c and LDL and cholesterol [13].

Unlike our results, few studies reported that patients with T2D and an HbA1c value greater or equal 7.0% showed a significant increase in TC and LDL levels without significant changes in TG and HDL level compared with the patients with T2D and an HbA1c value of <7.0% [14]. A study from Sudan showed no significant differences in TG, TC, LDL, and HDL levels between the glycemic control group and the uncontrolled group [15]. A study from Montenegro reported an association of high HbA1c levels with abnormal TG, TC, LDL, and HDL [16].

We also found a variation in HbA1c and dyslipidemia incidence in different age groups. Cholesterol level was insignificantly higher among patients with T2D aged 40-60 years than among the old age group (189.4 vs. 184.4 mg/dl; P = 0.627). HDL level among patients aged 20-39 years was 38.0 mg/dl compared to 52.3 mg/dl for patients aged >60 years with statistically significant difference (P = 0.002). TG was significantly higher among patients aged 20-29 years (210.6 mg/dl) than others aged >60 years (129 mg/dl) with P = 0.001. The average level of HbA1c among young patients (20-29 years) was 7.8% compared to 8.4% for patients aged >60 years (P = 0.186). We explain these findings because of the medications taken by the older age group. This study has a few limitations. First, we could not identify the co-morbidities, medications, and social history of the participants. We suggest further studies in the future on a larger population, considering the limitations in the current study.

## Conclusions

In the current study, we assessed the association between HbA1c levels and lipid profiles in patients with T2D at the KFU Health Center in the eastern region of the Kingdom of Saudi Arabia. Our results on the adjusted relationship of HbA1c with lipid profile through regression model have demonstrated that HDL alone had significant adjusted relation with HbA1c if other factors are kept constant. We suggest focusing on TC and HDL levels in T2D health management in future studies.
